# Exposure to digital marketing enhances young adults’ interest in energy drinks: An exploratory investigation

**DOI:** 10.1371/journal.pone.0171226

**Published:** 2017-02-02

**Authors:** Limin Buchanan, Bridget Kelly, Heather Yeatman

**Affiliations:** Early Start Research Institute, School of Health and Society, Faculty of Social Sciences, University of Wollongong, Wollongong, New South Wales, Australia; University of Cambridge, UNITED KINGDOM

## Abstract

Young adults experience faster weight gain and consume more unhealthy food than any other age groups. The impact of online food marketing on “digital native” young adults is unclear. This study examined the effects of online marketing on young adults’ consumption behaviours, using energy drinks as a case example. The elaboration likelihood model of persuasion was used as the theoretical basis. A pre-test post-test experimental research design was adopted using mixed-methods. Participants (aged 18–24) were randomly assigned to control or experimental groups (*N* = 30 each). Experimental group participants’ attitudes towards and intended purchase and consumption of energy drinks were examined via surveys and semi-structured interviews after their exposure to two popular energy drink brands’ websites and social media sites (exposure time 8 minutes). Exposure to digital marketing contents of energy drinks improved the experimental group participants’ attitudes towards and purchase and consumption intention of energy drinks. This study indicates the influential power of unhealthy online marketing on cognitively mature young adults. This study draws public health attentions to young adults, who to date have been less of a focus of researchers but are influenced by online food advertising.

## Introduction

Young adults are experiencing rapid weight gain [1–2] and are at greater risks of developing weight-related non-communicable diseases over time. Data from the Australian National Health Survey 2014–15 indicated that approximately 39% young adults aged 18–24 years were overweight or obese [3] and this age group consumed more unhealthy food and beverages than any other age groups [4]. Despite the growing health issues among this age group, research about influences on young adults’ consumption behaviours is scarce.

This study defined “young adults” as those aged 18 to 24 years, consistent with Australian National Health Survey 2014–15 classification and also previous publications on young Australians’ health-related behaviours [5–7]. The years from 18 to 24 years mark a distinct life stage. Some have defined this stage as “emerging adulthood”, a transitional stage between adolescence and full adult status [8]. Legally, they are adults, but they are still on the learning stage of becoming adults where many experience transitions from high school to work or college, from the parental home to their own accommodation, from passive meal recipients to first time food purchasers and preparers [9, 10]. The fact that this age group may face new challenges in life such as food purchase and preparation, but legally no one holds responsibilities over them, may open up new marketing opportunities for food and beverage industry. As indicated by Freeman et al. [10], the food industry may shift their promotional target from children to young adults in response to the increasing scrutiny over children’s unhealthy food marketing exposures.

Unhealthy food and beverage marketing towards young adults through the digital media is of particular relevance. This form of marketing is less scrutinised than the traditional marketing on television or in newspapers [11] but is pervasive for young adults who have grown up surrounded by digital technologies and have a strong online presence [12]. The more engaging and invasive forms of advertising on digital platforms, such as through social media sites and advergames, have been linked to unhealthy food consumption among children [13–14]. Such online advertising may also be appealing to young adults. It is unknown how the persuasive messages of online food marketing influence young adults, as it is often assumed they have developed cognitive capabilities to critically interpret the persuasive intent of marketers [15].

Unhealthy products that are particularly relevant to digital food marketing and young adults are energy drinks. Energy drinks are a new beverage category, defined as *“a non-alcoholic caffeine containing beverage typically consumed to provide an energy boost or for mental alertness”* [16]. Common examples of these drinks in the Australian market include Red Bull, Mother, V, Monster and Rockstar. Energy drinks were selected as a case example in this study due to a number of reasons. Firstly, consumption of energy drink is a public health concern. These drinks contain high amounts of caffeine and stimulants such as guarana, ginseng, and B groups vitamins, with varying amounts of sugar and protein, which can be linked to weight gain and a range of adverse health events such as headache and irritation [17–19]. A recent review conducted by Visram and colleagues [20] further suggested that the consumption of energy drinks are associated with risky behaviours, including sensation-seeking and self-destructive behaviours. Secondly, these drinks are marketed to improve alertness, concentration, and stamina [21] and may therefore be especially popular among young people, students and party-goers. Thirdly, energy drink brands predominantly utilise digital media for promotional purposes, for example, website, online games and social media platforms such as Facebook, Twitter, and YouTube [22]. Lastly, marketing of energy drinks is not strictly regulated in Australia. Energy drinks are regulated under the Food Standard Australia New Zealand (FSANZ) code in relation to product composition and labelling [23]. The advertising and marketing code established by the Australian Association of National Advertiser (AANA) does not cover marketing activities on digital platforms [24].

The mechanisms of the influences of digital marketing on food-related behaviours are not well understood. Marketing literature exploring tobacco, alcohol, and fast food products adopted the notion of marketing receptivity to understand consumers’ responses to marketing contents [25–27]. This approach suggested that consumers’ responses progress through several stages before any behaviour change. Responses range from low receptivity (exposure to marketing contents), to medium receptivity (attend to and understand the marketing contents), and then high receptivity (development of a cognitive or affective response to the marketed products or brands) [25–27]. Evidence of consumers’ receptivity to the marketing contents includes indications of positive cognitive or affective responses to the marketing contents or communications [25], such as a change in attitude towards the marketed product or brand [28]. Attitude changes have been recognised by numerous psychological theories as one of the intermediate mental effects of marketing exposure [28, 29].

The present study applied the concept of marketing receptivity to explore the possible causal pathway where exposure to digital food marketing may prompt a mental response (attitude change) as well as intermediate cued purchase and consumption intentions of the marketed brands and products. We hypothesised that exposure to digital marketing of energy drinks would enhance young adults’ attitudes towards, and intended purchase and consumption of, these drinks. Findings from our study indicated that digital food marketing has impacts on young adults’ attitude towards and their intended purchase and consumption of energy drinks.

## Materials and methods

This study adopted a pre-test post-test control group experimental model. A mixed-methods design was employed utilising a quantitative survey technique complemented by qualitative semi-structured interviews. All materials and procedures were approved by the University of Wollongong Human Research Ethics Committee (HE15-280). Participant Information Sheets were provided to the participants and their written consents were obtained before their participation in the study.

### Participants

Young adults aged 18 to 24 years were recruited in the Illawarra region of New South Wales, Australia, between July 2015 and March 2016. Recruitment included promoting the study through handing out flyers in university lectures and community colleges, at bus-stops, community centres, local sports clubs and a local theme park; posts and tweets on social media sites; and snowball sampling. The study was promoted as a study about Internet usage and food consumption. The advertisement did not make it explicit that it would be asking questions about energy drinks marketing in order to recruit both energy drinks users and non-users. Any young adults aged 18 to 24 years and knew how to use the Internet met the inclusion criteria. Individuals who were interested to participate in the study contacted the lead author via email or phone. Times and venues (all public spaces) were then arranged for the study at participants’ convenience. Participants were randomly assigned to either the control or experimental group. Randomisation was performed after the study sessions were booked; the lead author randomly picked out sealed envelopes that contained control or experimental surveys. Participants who completed the study were entered into a lucky draw of iTunes and Coles (major supermarket) vouchers of different values ($10-$50) as recognition for their time.

### Materials

#### Exposure experiment

The selected exposure materials to examine the impact of digital marketing on the participants in the experimental group were the websites and social media sites of two popular energy drinks brands: *Red Bull* and *V Energy*. These two brands were selected due to their popularity [30] and their distinctive marketing appeal strategies, as revealed in a previous content analysis of a range of energy drinks’ websites and social media sites [31]; *Red Bull* positions their brand as a high budget, sophisticated energy drink product by sponsoring high risk, expensive sports such as Formula 1 car racing, while *V Energy* used sophomoric humour such as superheroes to attract audiences. Control group participants were exposed to the websites and social media sites of two nut bars brands; *Carman’s* and *Go Natural*. An online search of beverage products for the healthy comparison was conducted but beverage options such as soft drinks, sports drinks and water products do not use online brand images promotion. The two nut bar brands were selected as they were relatively healthy food products in comparison to energy drinks and their use of positive brand images as marketing strategies was similar to the selected energy drink brands. All of the selected sites were publicly accessible. The researcher audited the sites every fortnight to ensure the exposed materials were consistent across the period between the first participant, who completed in July 2015, and the final participant, who completed in March 2016.

#### Pre- and post-test surveys

Experimental and control group participants self-completed the paper-based surveys at pre- (S1 Fig) and post-test (S2 and S3 Figs). Participants’ attitudes towards, and purchase intention of, the two test energy drinks brands and energy drinks products in general were measured at both time points. Nut bars, soft drinks and muesli bars brands were included in the surveys as distractors. Participants’ attitudes towards the energy drinks were measured by the mean of five 7-point semantic-differential scales; “bad/good”, “unfavourable/favourable”, “unappealing/appealing”, “likeable/unlikeable”, “pleasant/unpleasant”. This scale had previously been used by other studies to measure brand attitudes [32–33] and was derived from Batra and Stayman [34]. The internal consistency of these measures was (Cronbach Alpha Analysis) α = 0.941. Participants’ purchase intentions of energy drinks were measured by the widely used 5-point intention scale [35], ranging from “definitely will not purchase” to “definitely will purchase”. Demographic and Internet usage questions were also included in the pre-test survey.

#### Post-test semi-structured interview

The post-test quantitative survey was supplemented by a semi-structured interview (S4 and S5 Figs). The audio-recorded interviews were conducted by the lead author, who also wrote field notes during the interviews. Research into young adults’ beliefs and attitudes towards digital marketing of food and beverage products is scarce and hence the interview was used to provide insights into participants’ thoughts and feelings about the exposed materials, brands and energy drinks products in general. The interview questions were categorised into two main sections: (i) perceptions of brands, where participants’ were asked their views of the test energy drink brands, as well as the exposed websites and social media sites, and (ii) perception of energy drinks, where participants were asked about their own experiences and thoughts of energy drinks products and the consumption of these drinks among young people. Questions about nut bar products were included for the control group participants.

### Procedures

Participants took approximately 30 minutes to complete the study: pre-test survey (approximately 10 minutes), exposure experiment (fixed length- 8 minutes; 4 minutes on each brand), and post-test survey and semi-structured interview (approximately 10-15minutes). Participants filled in the pre-test survey before they were asked to browse the two selected energy drinks brands’ websites and social media sites (nut bar brands for control group). Participants were told to browse the sites freely using a laptop; they were allowed to browse whatever contents on the sites they chose, e.g. photos, videos, company information, advergames, recipes, as long as they stayed within the same website. After browsing the online materials, participants were asked to fill in the post-test survey and to participate in the interview. To measure participants’ food and beverage products preferences, participants were asked to select one food or drink item from a range of food and beverage products photos displayed on a poster before and after the experiment. To mimic a real-life ‘unsupervised’ web browsing scenario, the study was conducted at community settings (e.g., meeting rooms, cafes and parks) and participants were not ‘supervised’ during the study. Participants in the experimental group were given a Fact Sheet about the negative impacts of energy drinks consumption [36] after the completion of the study.

### Statistical analysis

#### Quantitative data analysis

A pilot study (*N* = 16) was conducted to pre-test the research instruments and to calculate the required sample size using G-Power statistical analysis software [37]. The input parameters entered into the software were effect size = .8, α = .05, and between-groups comparison effect size of attitude change towards energy drinks observed in the pilot study (d = .52). A total sample size of 60, 30 each in two groups, was needed to obtain statistical power of .92 level [38].

Statistical analysis was undertaken using the Statistical Package for Social Sciences (SPSS) for Windows, version 21. The internal consistency of the previously standardised attitude scale was examined by Cronbach Alpha Analysis. Based on data distribution, Independent sample t-tests or Mann-Whitney U tests were utilised to determine the changes in attitudes towards, and purchase intention of, energy drinks. Fisher-exact test was used to examine the changes in intended consumption of energy drinks before and after the experiment. *P* values of < 0.05 were considered statistically significant.

#### Qualitative data analysis

Qualitative data were analysed based on the field notes taken by the lead author during the interviews. The audio-recordings were used to check the completeness of written field notes as soon as the interviews were completed and while the reflections remained fresh [39]. The primary purpose of the semi-structured interview was to identify key factors which had led to the changes in attitudes measured after the experiment and verbatim transcription of the audio data was not undertaken. The benefits of using written field notes during the interview have been demonstrated by several researchers [39–40]; not only that it may improve the efficiency, it may also ease the difficulties of data coding. Content analysis [41] was then conducted to elicit common themes arising from the participants’ responses about their perceptions of the energy drinks brands as well as the exposed online materials. Content analysis involved: (i) open coding where the written notes were read through several times and (ii) grouping and categorisation where the headings were assigned to related contents before they were grouped into higher order categories [41]. The co-author who was not involved in the data collection reviewed the established themes and the respective responses. The established themes were categorised based on the elaboration likelihood model (ELM) of persuasion as discussed below.

### Theoretical framework

The elaboration likelihood model (ELM) of persuasion as proposed by Cacioppo and Petty [42] has particular relevance to the examination of food marketing impacts. Various studies have used ELM to understand the influences of product advertising [43–45]. ELM is a framework that describes the thinking processes that might occur when an individual is exposed to persuasive communication. ELM highlights dual routes of information processing, central and peripheral, that may lead to changes in an individual’s attitudes and behaviours [44–45]. In the central route, an individual engages in high level information processing, that is, he/she carefully thinks about the persuasive message. An example of a central cue is the nutrition claims of a food product. In contrast, a peripheral route involves low level of information processing. An individual processes the message without much cognitive effort. A common peripheral cue used by advertisers is emotional appeals, such as fun and happiness [45]. The more pertinent element of ELM in this study was anticipated to be the peripheral route, as previous analyses of the digital marketing contents of energy drinks brands [31] have found that the energy drinks industry steered away from actual product promotions/information and used emotional appeals like excitement to engage young adults.

## Results

Participants in the experimental and control groups were similar (statistically) in gender, age, education levels, Internet usage, and their usual Internet activities (Table 1). Although attempts were made to recruit participants from various demographic backgrounds, the majority of the study participants were university students (*N* = 49/60). At pre-test, participants’ attitudes towards, and purchase intention of, the test energy drinks brands and energy drinks products in general were similar between experimental and control groups (Table 1).

**Table 1:** Characteristics of study participants at pre-test.

Characteristics	Experimental (*N* = 30)	Control (*N* = 30)	*P* value	Total (*N* = 60)
Gender ^a^			.194	
Male	16	11		27
Female	14	19		33
Age ^b^, *Mean yr*	20	20	.892	20
Education level ^c^			.643	
High school or equivalent	3	4		7
TAFE qualification or equivalent	3	5		8
Bachelor’s degree	21	17		38
Postgraduate qualification	3	4		7
Internet usage ^c^			.068	
Several times a week	2	1		3
Every day	13	7		20
Several times a day	15	22		37
Usual Internet activity ^c^				
Emails	28	26	.977	54
Online games	9	10	.783	19
Facebook	28	28	1.000	56
YouTube	23	24	.756	47
Twitter	6	4	.492	10
Online shopping	16	17	.442	29
News	15	17	.608	22
Attitude^b^ *Mean± SD*				
Red Bull	- 0.5 ± 1.4	- 0.5 ± 1.7	.947	- 0.5 ± 1.5
V Energy	- 0.5 ± 1.4	- 0.8 ± 1.4	.402	- 0.7 ± 1.4
Energy drink products (general)	- 0.4 ± 1.5	- 0.5 ± 1.8	.824	- 0.5 ± 1.6
Purchase Intention^c^ *Median (mode)*				
Red Bull	-1.0 (-2.0)	-1.0 (-2.0)	.588	-1.0 (-2.0)
V Energy	-1.0 (-2.0)	-1.5 (-2.0)	.753	-1.0 (-2.0)
Energy drink products (general)	-1.0 (-2.0)	-1.0 (-2.0)	.545	-1.0 (-2.0)

^a^Pearson Chi-square test

^b^Independent sample t-test

^c^Mann-Whitney U test

### Attitudes and purchase intention

Effect sizes of the study were calculated based on the attitudes changed; the between group effect size was *d* = 0.71 while the within group effect was *d* = 0.55. Both were medium effect sizes [38].

Participants in the experimental group had significantly better attitudes towards the two test energy drink brands *Red Bull* (*t*(42) = -4.1, *p* = .000) and V Energy (*t*(53) = -3.5, *p* = .001), as well as energy drinks products in general (*t*(50) = -4.5, *p* = .000) than the control group participants after the experiment. After the experiment, participants in the experimental group compared with control group participants also showed significantly greater purchase intention of the two test energy drinks brands *Red Bull* (*U* = 222.5, *p* = .000) and *V Energy* (*U* = 243.5, *p* = .000), as well as energy drinks products in general (*U* = 395.5, *p* = .300) (Table 2).

**Table 2:** Comparisons of participants’ attitudes towards and purchase intention of, energy drinks after the experiment.

Measures	Post-test–Pre-test (*N* = 60)	Test result
Attitude^a^ *Mean ± SD*		
Red Bull	0.3 ± 1.0	*t*(42) = -4.1, *p* = .000*
V Energy	0.3 ± 1.1	*t*(53) = -3.5, *p* = .001*
Energy drinks products (general)	0.2 ± 0.8	*t*(50) = -4.5, *p* = .000*
Purchase Intention^b^ *Median (Interquartile range)*		
Red Bull	0.0 (0.0–1.0)	*U* = 222.5, *p* = .000*
V Energy	0.0 (0.0–1.0)	*U* = 243.5, *p* = .000*
Energy drinks products (general)	0.0 (0.0–1.0)	*U* = 395.5, *p* = .300*

*Significantly different when *p* < .05

^a^Independent- samples t-test

^b^Mann-Whitney U Test

Between group comparisons showed that at post-test, the participants in the experimental group showed significantly more positive attitudes towards the two test energy drink brands (*Red Bull*’s mean difference of an average of 5 x 7-point semantic scales = 1.0±1.8, *p* = .038; *V Energy*’s mean difference = 1.2±1.6, *p* = .006) and energy drinks products in general (mean difference = 1.0±1.6, *p* = .028), and slightly greater purchase intention of *V Energy* (Z = -2.01, *p* = .044) as compared to the control group (Table 3).

**Table 3:** Between group comparisons at post-test.

	Brand/Product	Experimental	Control	*P* value
**Attitude**^b^(5 item; -3 to 3)*Mean ± SD*	Red Bull	0.3 ± 1.7	- 0.7 ± 1.8	.038*
	V Energy	0.2 ± 1.7	- 1.0 ± 1.5	.006*
	Energy drink products (general)	0.2 ± 1.5	- 0.8 ± 1.7	.028*
**Purchase intention**^a^(-2 to 2)*Median (mode)*	Red Bull	0.0 (-2.0)	-1.0 (-2.0)	.087
	V Energy	-0.5 (-2.0)	-2.0 (-2.0)	.044*
	Energy drink products (general)	-1.0 (1.0)	-1.5 (-2.0)	.108

*Significantly different when *p* < .05

^a^ Mann-Whitney U Test

^b^ Independent- samples t-test

Attitudes of the experimental group participants towards the two test energy drink brands (*Red Bull*’s mean difference = 0.8±1.5, *p* = .001; *V Energy*’s mean difference 0.8±1.6, *p* = .001), as well as energy drink products in general (mean difference = 0.6±1.5, *p* = .000) were all significantly improved after the experiment (Table 4). Participants’ average attitude scale values all moved from the negative to positive ends. Although the experimental group participants’ purchase intentions towards the energy drinks remained negative, their purchase intentions of the two test energy drink brands (*Red Bull* Z = -2.724, *p* = .006; *V Energy* Z = -3.000, *p* = .003) were improved statistically significantly after the test. Surprisingly, control group attitudes towards energy drinks products in general, and their purchase intention towards the two energy drinks brands were significantly worsened at post-test although they were not exposed to any energy drinks materials. As a side note, control group participants showed better attitude towards and greater purchase intention towards one of the test nut bar brands.

**Table 4:** Within group comparisons.

Experimental				
	Brand/Product	Pre-test	Post-test	*P* value
**Attitude** ^b^(5 item; -3 to 3)*Mean ± SD*	Red Bull	- 0.5 ± 1.4	0.3 ± 1.7	.001*
	V Energy	- 0.5 ± 1.4	0.2 ± 1.7	.001*
	Energy drink products (general)	- 0.4 ± 1.5	0.2 ± 1.5	.000*
**Purchase intention** ^a^(-2 to 2)*Median (mode)*	Red Bull	-1.0 (-2.0)	0.0 (-2.0)	.006*
	V Energy	-1.0 (-2.0)	-0.5 (-2.0)	.003*
	Energy drink products (general)	-1.0 (-2.0)	-1.0 (1.0)	.223
**Control**				
**Attitude** ^b^(5 item; -3 to 3)*Mean ± SD*	Red Bull	- 0.5 ± 1.7	- 0.7 ± 1.8	.129
	V Energy	- 0.8 ± 1.4	- 1.0 ± 1.5	.447
	Energy drink products (general)	- 0.5 ± 1.8	- 0.8 ± 1.7	.036*
	Carmans’ nut bar	1.0 ± 1.3	1.8 ± 1.0	.000*
	Go Natural’s nut bar	0.7 ± 1.2	1.2 ± 1.2	.068
	Nut bar products (general)	1.2 ± 1.1	1.4 ± 1.0	.108
**Purchase intention** ^a^(-2 to 2)*Median (Mode)*	Red Bull	-1.0 (-2.0)	-1.0 (-2.0)	.046*
	V Energy	-1.5 (-2.0)	-2.0 (-2.0)	.046*
	Energy drink products (general)	-1.0 (-2.0)	-1.5 (-2.0)	.589
	Carmans’ nut bar	1.0 (1.0)	1.0 (1.0)	.003*
	Go Natural’s nut bar	1.0 (1.0)	1.0 (1.0)	.951
	Nut bar products (general)	1.0 (1.0)	1.0 (1.0)	.052

*Significantly different when *p* < .05

^a^ Wilconxon- Signed Rank Test

^b^ Paired-samples t-test

### Consumption intention

The participants involved in the exposure experiment had significantly enhanced intention to consume energy drinks at post-test. Seven participants showed positive change, from no intention to consume energy drinks at pre-test to intended to consume energy drinks at post-test, χ^2^(1) = 7.9, *p* = .005 (Table 5). The number of participants in the experimental group who selected energy drinks from the poster rose from four to eleven after the experiment. Control group participants had greater intention to consume nut bar products after the experiment χ^2^(1) = 16.6, *p* = .000.

**Table 5:** Participants’ intended consumption of the energy drinks and nut bar products after the experiment.

Consumption Intention^a^ *Count*	Experimental(*N* = 30)	Control (*N* = 30)	Positive change from pre-test to post-test^b^ (*N* = 60)	Test result
Energy drinks products (general)				
Pre-test	4	2		
Post-test	11	0	7	χ^2^(1) = 7.9, *p* = .005*
Nut bars products (general)				
Pre-test	4	6		
Post-test	2	19	13	χ^2^(1) = 16.6, *p* = .000*

*Significantly different when *p* < .05

^a^Fisher’s exact Test

^b^Weight cases command was conducted before chi-square analysis on a contingency table. Due to the small sample size, Fisher’s exact Test was used.

### Qualitative interview findings

#### Appealing factors of the exposed online materials and brands

**Fig 1:**
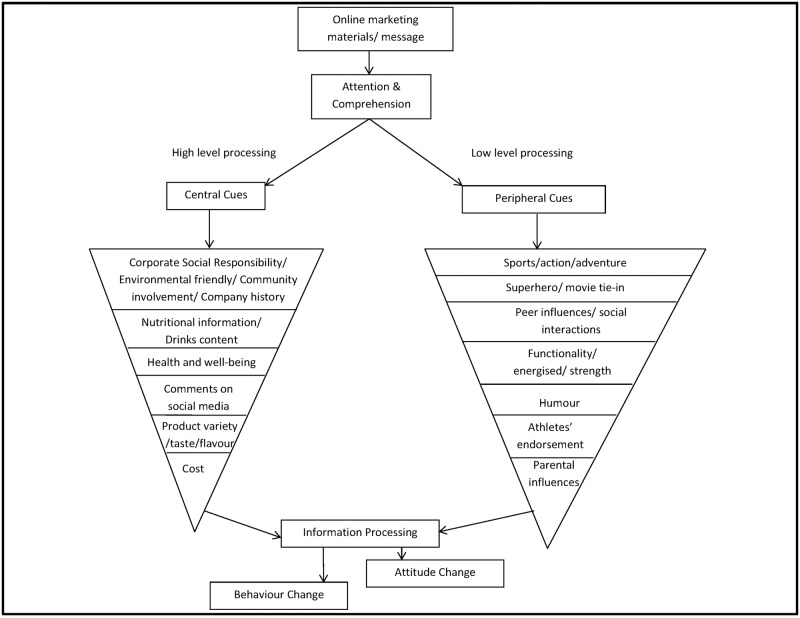
Qualitative findings using ELM as a theoretical basis.

Consistent with the quantitative survey findings, a number of participants in the experimental group reported the exposure to the online materials had somewhat improved their feelings towards the test brands, with the majority of the participants showing more positive attitudes towards at least one of the test energy drink brands. A range of appealing elements (Fig 1) on the test brands digital platforms changed participants’ attitudes towards these brands. The most widely mentioned factors were the corporate social responsibility initiatives reported by the brands, community involvement and sponsorship from *Red Bull*. For instance one participant *(participant a*, *male*, *18 years)* noted,

*“Feel like they are trying to do good stuff for the environment; their cans are 100% recycled, and minimise transportation…that’s a good thing, also they sponsored a lot of sports…”*.

Both brands listed their drink products’ nutritional information on their websites. The majority of participants favoured the “honesty” of the brands, for example,

“*V showed their nutrient contents*, *I was surprised by the low guarana content*, *it is not shady at all*, *really appealing”*.

(participant b, male, 18 years)

Only a minority of the participants took a more critical approach towards the nutritional claims made by the brands,

*“I looked at the ingredients of both brands*, *so superficial*, *no scientific proof benefits… some of them are just a sentence*, *don’t believe that it will help me to concentrate at all”*.

(participant c, male, 22 years)

The superhero theme of V Energy appeared to be a selling point for some participants. *“I am into Avengers (movie) that would convince me to purchase V over Red Bull…” (participant d*, *female*, *18 years)*. The majority of the participants visited the test brands’ social media sites (mainly Facebook) during the experiment. The comments that were posted from other Facebook users seemed to have greater influence on the participants than posts by the companies;

*“V’s Facebook page was filled with positive comments from the others and the website was more engaging (not just about sports)…”*, *“Red Bull’s Facebook page made it even more unappealing*, *a lot of people left negative comments on their Facebook page…”*

*(participant e, female, 22 years)*.

#### Views on energy drinks

Relatively few frequent energy drinks users participated in the study.

The majority of the participants reported they had their first energy drinks when they were teenagers, usually under peer influence,

“I first tried energy drinks in year8 or 9, friends bought me the drink, felt cool to drink it, we were under 18, can’t drink alcohol, so it was the next best thing…”

*(participant f*, *female*, *18 years)*.

Although most of the participants reported they had ‘grown out of’ / no longer consumed these drinks, many reported consuming energy drinks for functionality purposes, such as for stamina for assignments, computer games, shift work and parties. Almost all participants were aware of the ‘practice’ of mixing alcohol with energy drinks and many reported they had consumed these drinks when going out parties with friends, despite reporting they knew the drinks were ‘bad’ for them.

## Discussion

This study demonstrated the potential influential power of food and beverage digital marketing on cognitively mature young adults. After a short exposure to digital marketing materials, participants had a better impression of, greater purchase intention and were more likely to consume energy drinks. Of surprise was the revelation that central cues, such as corporate social responsibility as demonstrated by the brands, was reported by the participants in a manner that implied they had greater impacts on the young adults than the peripheral cues like emotional appeals.

The positive association between digital marketing and attitudes towards the beverage brands observed in this study contributes new insights into the influences of online environments on young adults’ food-related behaviour. There have been a growing number of studies reporting on the use of digital platforms for marketing that have established the association between online alcohol marketing and consumption behaviours [46–50]. These studies, however, were cross-sectional designs and could not determine the direction of causality. There was one controlled intervention where the authors compared the effects of alcohol marketing online between states in the United States of America (USA) that do or do not have bans on advertising alcohol. This study found digital marketing of alcohol reduced the effectiveness of alcohol advertising bans [51], which indicated the influential power of online marketing materials on consumers’ attitudes towards food products. Previous studies on alcohol have argued that exposure to marketing contents enhanced consumers’ receptivity to alcohol marketing [28, 47]. Although not the same product category, our findings contribute to this literature showing how young adults may become receptive to risky product marketing.

The study reported here found that young adults’ attitudes towards, purchase and consumption intentions of the brands were influenced by digital marketing. Online content that was most highly reported to have influenced their attitudes towards the brands were linked to central information processing cues, that information cognitively elaborated through the central route, as described in the ELM framework. There is a general assumption that young adults are cognitively mature and possess the ability to understand the persuasive intention of marketers [52]. However, our findings suggest that although young adults realised the online materials were designed to promote energy drinks products, they were not necessarily capable to defend themselves against marketing content such as the brands’ community involvement, contribution to charities, and environmentally friendly efforts (i.e. corporate social responsibility). Young adults also valued the ‘honesty’ of the brands by declaring the contents of their drink.

The ability of these central cues to distort the promotion intentions of the online marketing among young adults was unexpected. Previous assessments of the content of food advertising, especially among children, has generally found that emotionally evocative peripheral cues in television advertisements were used to attract audiences [45, 53]. Our experiment only included a short exposure and the improvements in attitudes towards brands may only have a short lasting effect. However, this finding was significant since central cues are believed to have more profound impact on attitude changes and more predictive of future behaviour than the peripheral cues [42]. Additionally, participants in this study were mainly educated young adults who reported to be aware of the health problems of energy drinks products. The impact of online marketing could be greater among young adults who are less educated or less health conscious.

The classification of central cues in this study was based on the assumption that participants may take more time to evaluate information such as the brands’ contributions to the environment. Although literature on corporate image advertising has previously discussed the notion of the processing of corporate social responsibility messages from the ELM insights [54–55], none have explored whether these messages are evaluated through the central or peripheral route. Participants’ pre-existing attitudes and personal values towards corporate social responsibility issues may influence how they assess these messages [56]. For instance, an individual who does not favour corporate social responsibility work may automatically process the message as sceptical (peripheral route to persuasion) whereas an individual who favours the notion of corporate social responsibility may scrutinise the information further (central route to persuasion) [55]. Given that the corporate social responsibility practices portrayed on the websites were mainly written contents and required participants’ thoughtful assessment, corporate social responsibility practices were classified as central cues in this study.

The interviews in this study also revealed other factors that contributed to young adults’ consumption of energy drinks, including peer influences and social opportunities involving mixing alcohol with energy drinks at parties. It was unknown whether these factors were induced by the peripheral cues of the online marketing messages (e.g. energised, fun). Many participants reported to have first started consuming energy drinks during adolescence. Future studies may need to include participants from a younger age group.

Although not the main focus of this study, control group participants showed more positive feelings, greater purchase and consumption intentions towards one of the nut bar brands. Oddly, they also reported more negative attitudes towards the energy drinks brands although they were not exposed to any energy drinks related online materials. One possible explanation for this was that the healthy messages delivered on the nut bar brands sites may have counteracted participants’ desire to have unhealthy products. This unexpected finding highlighted a potential avenue for public health intervention. Positive brand image portrayed by the food companies may be effective in increasing healthy food consumption while reducing unhealthy food consumption. Future studies may explore further on this.

### Strengths and limitations

The strengths of this study included the use of qualitative interviews to supplement the findings of quantitative surveys, and the application of ELM as a theoretical framework to better understand the variables that might have influenced young adults during the online exposure. Our pre-test post-test control group experimental trial was sufficiently powered and statistically significant changes were found on participants’ attitudes, purchase and consumption intentions. However, these findings need to be interpreted with caution since the regional, non-representative convenience sample may limit the generalisability of our findings. Recruitment of young people from lower socio-economic backgrounds remains a challenge but the impact of online unhealthy food marketing on this cohort warrants further investigation. The medium effect size of this study was in accordance with other research on attitudes towards food advertising where small to moderate effect sizes were found [57–58]. Participants in the control group were more likely to be females and have higher Internet usage. These differences were not statistically significant but could potentially influence the findings, for example, female participants may have different responses to the macho image (extreme sports) of energy drinks brands. Another limitation of the study was that participants were given a task to browse the sites during the experiment and thus may have paid more attention than normal to the information presented online. This might explain the highly reported processing of central cues in this study. This could also potentially introduce bias if the participants felt that the researcher anticipated some changes at post-test, although the true motive of the study was masked. As pointed out by other relevant advertising literature, digital marketing exposure is only one of the many factors that influence consumers’ receptivity to the marketing contents [26, 29]. Further studies are required to explore other precursors or influences that lead to young adults’ consumption of risky food or beverage products.

## Conclusions

With the greater interactions of young people with online environments and social media, it is important to understand how young people’s consumption patterns and health behaviours may be affected. This study provides useful insights into the online environment that may contribute to unhealthy behaviours of young adults. Greater understanding of the types of cues and their influences on young adults’ attitudes and potentially also their behaviours can inform professional practice and regulatory policies relating to online environments.

## Supporting information

S1 Fig Pre-test survey. Self-administered survey for both the experimental and control groups’ participants before the exposure experiment.
(DOC)

S2 Fig Post-test survey (experimental group). Self-administered survey for experimental group participants after the exposure experiment.
(DOCX)

S3 Fig Pos-test survey (control group). Self-administered survey for control group participants after the exposure experiment.
(DOCX)

S4 Fig Post-test interview guide (experimental group). Semi-structured interview guide for the experimental group participants after the exposure experiment.
(DOCX)

S5 Fig Post-test interview guide (control group). Semi-structured interview guide for the control group participants after the exposure experiment.
(DOCX)

S1 File Minimal dataset. Minimal dataset obtained from pre- and post-test surveys.
(XLSX)

## References

[1] Allman-Farinelli MA, Chey T, Bauman AE, Gill T, James WPT. Age, period and birth cohort effects on prevalence of overweight and obesity in Australian adults from 1990 to 2000. European Journal Of Clinical Nutrition. 2008;62(7):898–907. 17440514 10.1038/sj.ejcn.1602769

[2] Nikolaou CK, Hankey CR, Lean MEJ. Weight changes in young adults: a mixed-methods study. International Journal of Obesity. 2015;39(3):508–13. 10.1038/ijo.2014.160 25152239

[3] Australian Bureau of Statistics (ABS). 4364.0.55.001—National Health Survey: First Results, 2014–15 Canberra, Australia: Australian Bureau of Statistics. 2015. http://www.abs.gov.au/AUSSTATS/abs@.nsf/DetailsPage/4364.0.55.0012014-15?OpenDocument.

[4] Australian Bureau of Statistics (ABS). 4364.0.55.007—Australian Health Survey: Nutrition First Results—Foods and Nutrients, 2011–12. 2014. http://www.abs.gov.au/ausstats/abs@.nsf/Lookup/by%20Subject/4364.0.55.007~2011-12~Media%20Release~Soft%20drink,%20burgers%20and%20chips%20-%20the%20diet%20of%20our%20young%20males%20(Media%20Release)~1.

[5] Perez DA, Grunseit AC, Rissel C, Kite J, Cotter T, Dunlop S, et al Tobacco promotion 'below-the-line': exposure among adolescents and young adults in NSW, Australia. BMC Public Health. 2012;12:1–9. 22691578 10.1186/1471-2458-12-429 PMC3391174

[6] Miller CL, Hickling JA. Phased-in smoke-free workplace laws: Reported impact on bar patronage and smoking, particularly among young adults in South Australia. Australian and New Zealand Journal of Public Health. 2006;30(4):325–327. 16956160 10.1111/j.1467-842x.2006.tb00843.x

[7] Piggford T, Raciti M, Harker D, Harker M. The influence of residence on young adult attitudes toward healthy eating. Social Marketing Quarterly. 2008;14(2):33–49.

[8] Arnett J. Emerging Adulthood: The Winding Road from the Late Teens Throught the Twenties. New York, NY: Oxford University Press; 2004.

[9] Blatterer H. The Changing Semantics of Youth and Adulthood. Cultural Sociology. 2010;4(1):63–79.

[10] Freeman B, Kelly B, Vandevijvere S, Baur L. Young adults: beloved by food and drink marketers and forgotten by public health? Health Promotion International. 2015;17(7):856–865. 10.1093/heapro/dav081 26276799

[11] Montgomery KC, Chester J, Grier SA, Dorfman L. The New Threat of Digital Marketing. Pediatric Clinics of North America. 2012;59(3):659–675. 22643172 10.1016/j.pcl.2012.03.022

[12] Montgomery KC, Chester J. Interactive Food and Beverage Marketing: Targeting Adolescents in the Digital Age. Journal of Adolescent Health. 2009;45(3 SUPPL.):S18–S29. 10.1016/j.jadohealth.2009.04.006 19699433

[13] Alvy LM, Calvert SL. Food Marketing on Popular Children’s Web Sites: A Content Analysis. Journal of the American Dietetic Association. 2008;108(4):710–3. 10.1016/j.jada.2008.01.006 18375231

[14] Kelly B, Bochynska K, Kornman K, Chapman K. Internet food marketing on popular children's websites and food product websites in Australia. Australian and New Zealand Journal of Public Health. 2008;32:522–8. 18298882 10.1017/S1368980008001778

[15] Baumgartner E. Affective responses to movie posters: Differences between adolescents and young adults. International journal of psychology. 2012;47(2):154–160. 10.1080/00207594.2011.597398 22046997

[16] Food Regulation Standing Committee Caffeine Working Group. Food Regulation Policy Options Paper- the Regulation of Caffeine in Foods. 2013.

[17] Heckman MA, Sherry K, de Mejia EG. Energy drinks: An assessment of their market size, consumer demographics, ingredient profile, functionality, and regulations in the United States. Comprehensive Reviews in Food Science and Food Safety. 2010;9(3):303–17. 10.1111/j.1541-4337.2010.00111.x 33467819

[18] Huhtinen H, Lindfors P, Rimpelä A. Adolescents’ use of energy drinks and caffeine induced health complaints in Finland. Arja Rimpelä. 2013;23(suppl 1).

[19] Schwartz DL, Gilstad-Hayden K, Carroll-Scott A, Grilo SA, Ickovics JR, McCaslin C, et al Energy drinks and youth self-reported hyperactivity/inattention symptoms. Academic Pediatrics. 2015;15(3):297–304. 10.1016/j.acap.2014.11.006 25676784 PMC4772143

[20] Visram S, Crossley SJ, Lake AA, Cheetham M, Riby DM. Consumption of energy drinks by children and young people: A rapid review examining evidence of physical effects and consumer attitudes. BMJ Open. 2016;6(10):1–23. 10.1136/bmjopen-2015-010380 PMC5073652 27855083

[21] Fogger S, McGuinness TM. Update on energy drinks and youth. Journal of Psychosocial Nursing and Mental Health Services. 2011;49(12):17–19. 10.3928/02793695-20111102-03 22085613

[22] Yale Rudd Center for Food Policy & Obesity. Sugary drink FACTS: Evaluating sugary drink nutrition and marketing to youth. 2011. http://www.sugarydrinkfacts.org/resources/sugarydrinkfacts_report.pdf.

[23] Food Standard Australia New Zealand. Standard 2.6.4 Formulated Caffeinated Beverages. 2013. http://www.comlaw.gov.au/Details/F2013L00050.

[24] Australian Beverages Council. Energy Drinks- An Industry Commitment. 2011. http://australianbeverages.org/wp-content/uploads/2013/04/EnergyDrinks_AnIndustryCommitment.pdf.

[25] Pierce J, Choi W, Gilpin E, Farkas A, Berry C. Tobacco Industry Promotion of Cigarettes and Adolescent Smoking American Medical Association. 1998;279(7):511–516. 10.1001/jama.279.7.511 9480360

[26] McClure AC, Stoolmiller M, Tanski SE, Engels R, Sargent JD. Alcohol Marketing Receptivity, Marketing-Specific Cognitions, and Underage Binge Drinking. Alcoholism-Clinical and Experimental Research. 2013;37:E404–E13. 10.1111/j.1530-0277.2012.01932.x PMC3548023 23256927

[27] McClure AC, Tanski SE, Gilbert-Diamond D, Adachi-Mejia AM, Li Z, Li Z, et al Research Article: Receptivity to Television Fast-Food Restaurant Marketing and Obesity Among U.S. Youth. American Journal of Preventive Medicine. 2013;45:560–568. 10.1016/j.amepre.2013.06.011 24139768 PMC3934414

[28] Morgenstern M, Isensee B, Hanewinkel R, Sargent JD. Attitudes as mediators of the longitudinal association between alcohol advertising and youth drinking. Archives of Pediatrics and Adolescent Medicine. 2011;165(7):610–616. 10.1001/archpediatrics.2011.12 21383258

[29] Austin EW, Chen M-J, Grube JW. Original article: How does alcohol advertising influence underage drinking? The role of desirability, identification and skepticism. Journal of Adolescent Health. 2006;38:376–384. 10.1016/j.jadohealth.2005.08.017 16549298

[30] The Australian. Popular energy drinks have the majors buzzing. 2011. http://www.theaustralian.com.au/business/popular-energy-drinks-have-the-majors-buzzing/story-e6frg8zx-1225985880656.

[31] Buchanan L, Kelly B, Yeatman H, Kariippanon K. Digital marketing and young adults: A content analysis of energy drinks’ digital media platforms "Forthcoming"

[32] Bellman S, Potter RF, Treleaven-Hassard S, Robinson JA, Varan D. The Effectiveness of Branded Mobile Phone Apps. Journal of Interactive Marketing. 2011;25:191–200.

[33] Ballouli J, Hutchinson M. Effects of Brand Music on Attitudes toward a Team Advertisement. Journal of Issues in Intercollegiate Athletics. 2013;6:268–285.

[34] Batra R, Stayman DM. The Role of Mood in Advertising Effectiveness. Journal of Consumer Research. 1990;17(2):203–214.

[35] Jamieson LF, Bass FM. Adjusting Stated Intention Measures to Predict Trial Purchase of New Products: A Comparison of Models and Methods. Journal of Marketing Research (JMR). 1989;26(3):336–345.

[36] Australia Drug Foundation. Energy drinks: do they really give you wings?. 2012. http://www.druginfo.adf.org.au/images/810_ADF_Factsheet_energy_web2012.pdf.

[37] Heinrich-Heine-Universität Düsseldorf. G*Power: Statistical Power Analyses for Windows and Mac. 2016. http://www.gpower.hhu.de/en.html.

[38] Cohen J. A Power Primer. Psychological Bulletin. 1992;112(1):155–159. 19565683 10.1037//0033-2909.112.1.155

[39] Halcomb EJ, Davidson PM. Is verbatim transcription of interview data always necessary?. Applied Nursing Research. 2006(19):38–42. 16455440 10.1016/j.apnr.2005.06.001

[40] Fasick FA. Some Uses of Untranscribed Tape Recordings in Survey Research. Public Opinion Quarterly. 1977;41(4):549–552.

[41] Elo S, Kyngas H. The qualitative content analysis process. Journal of Advanced Nursing. 2007;62(1):107–115. 10.1111/j.1365-2648.2007.04569.x 18352969

[42] Cacioppo J, Petty R. The Elaboration Likelihood Model of Persuasion. Advances in Consumer Research. 1984;11:673–675.

[43] Moore ES, Rideout VJ. The Online marketing of food to children: Is it just fun and games? Journal of Public Policy & Marketing. 2007;26(2):202–220.

[44] Bhutada NS, Menon AM, Deshpande AD, Perri M. Impact of Celebrity Pitch in Direct-to-Consumer Advertising of Prescription Drugs. Health Marketing Quarterly. 2012;29(1):35–48. 10.1080/07359683.2012.652576 22416924

[45] Kim H, Lee D, Hong Y, Ahn J, Lee K-Y. A content analysis of television food advertising to children: comparing low and general-nutrition food. International Journal of Consumer Studies. 2016;40(2):201–210.

[46] McClure A, Stoolmiller M, Tanski SE, Engels R, Sargent JD. Alcohol Marketng Receptivity, Marketing-Specific Cognitions, and Underage Binge Drinking. 2013;37:E404–E413. 10.1111/j.1530-0277.2012.01932.x PMC3548023 23256927

[47] McClure A, Tanski S, Li Z, Jackson K, Morgenstern M, Li Z, et al Internet alcohol marketing and underage alcohol use. Pediatrics. 2016;137(2):1–14. 10.1542/peds.2015-2149 PMC5011761 26738886

[48] Jones SC, Magee CA. Exposure to Alcohol Advertising and Alcohol Consumption among Australian Adolescents. Alcohol and Alcoholism. 2011;46(5):630–7. 10.1093/alcalc/agr080 21733835

[49] Hoffman EW, Pinkleton BE, Austin EW, Reyes-Velazquez W. Exploring College Students' Use of General and Alcohol-Related Social Media and Their Associations With Alcohol-Related Behaviors. Journal of American College Health. 2014;62(5):328–335. 10.1080/07448481.2014.902837 24635485

[50] Gordon R, Harris F, Marie MacKintosh A, Moodie C. Assessing the cumulative impact of alcohol marketing on young people's drinking: Cross-sectional data findings. Addiction Research and Theory. 2011;19(1):66–75.

[51] Goldfarb A, Tucker C. Advertising Bans and the Substitutability of Online and Offline Advertising. Journal of Marketing Research (JMR). 2011;48(2):207–27.

[52] Cornish LS. 'Mum, can I play on the internet?' Parents' understanding, perception and responses to online advertising designed for children. International Journal of Advertising. 2014;33(3):437–73.

[53] Warren R, Wicks RH, Wicks JL, Fosu I, Chung D. Food and Beverage Advertising on U.S. Television: A Comparison of Child-Targeted Versus General Audience Commercials. Journal of Broadcasting & Electronic Media. 2008;52(2):231–46.

[54] BöGel PM. Processing of CSR communication: insights from the ELM. Corporate Communications: An International Journal. 2015;20(2):128–143.

[55] Alan P, Lester WJ. Advertising corporate social responsibility initiatives to communicate corporate image: Inhibiting scepticism to enhance persuasion. Corporate Communications: An International Journal. 2009;14(4):420–439.

[56] Wang A, Anderson R. A Multi-Staged Model of Consumer Responses to CSR Communications. The Journal of Corporate Citizenship. 2011(41):50.

[57] Paek HJ, Hove T, Yoon HJ. Not all nutrition claims are perceived equal: Anchoring effects and moderating mechanisms in food advertising. Health Communication. 2011;26(2):159–170. 10.1080/10410236.2010.544281 21308579

[58] Dixon HG, Scully ML, Wakefield MA, White VM, Crawford DA. The effects of television advertisements for junk food versus nutritious food on children's food attitudes and preferences. Social Science & Medicine. 2007;65:1311–1123. 17587474 10.1016/j.socscimed.2007.05.011

