# Supplementary material for: Exposure to digital marketing enhances young adults’ interest in energy drinks: An exploratory investigation
Source: PLoS One. 2017 Feb 2;12(2):e0171226. doi: 10.1371/journal.pone.0171226 (PMC5289551; doi:10.1371/journal.pone.0171226)
Supplement: S1 Fig — Self-administered survey for both the experimental and control groups’ participants before the exposure experiment. (DOC) [file pone.0171226.s001.doc]

*Participant Number:* _____________________ *Date: ____________*

**PRE-TEST SURVEY**

**This questionnaire is part of a study of food-related Internet sites. Your participation in this study is voluntary and anonymous.**

1. Gender:

☐ Male

☐ Female

1. Age: ___________ Years
2. What is the highest level of education you have completed or are currently undertaking?

☐ None

☐ Primary school

☐ High school or equivalent

☐ TAFE qualification or equivalent

☐ Bachelor’s degree

☐ Postgraduate qualification

☐ Other, please specify __________________

1. If you are a university or TAFE student, what course/s are you currently undertaking?

___________________________________

1. How often do you access the Internet?

☐ Once a month or less

☐ Once a week

☐ Several times a week

☐ Every day

☐ Several times a day

1. Each time you get online, approximately how much time do you spend on the Internet?

☐ Less than 15 minutes

☐ 15 minutes to less than 30 minutes

☐ 30 minutes to less than one hour

☐ More than one hour

1. When you access the Internet, which of the following do you usually do? (can select more than one answer)

☐ Check or send emails

☐ Play online games

☐ Download music or video

☐ Use chat rooms

☐ Use web for school or work purposes

☐ Log on to Facebook

☐ Watch video on YouTube

☐ Check Tweets or use Twitter

☐ Online shopping

☐ Read news

☐ Use other computer applications. Please specify ________________________________

1. Please circle the number which seems most close to how you would describe:

| V Energy | Never heard of it  ☐ | Bad | 1 | 2 | 3 | 4 | 5 | 6 | 7 | Good |
| --- | --- | --- | --- | --- | --- | --- | --- | --- | --- | --- |
|  |  | Unfavourable | 1 | 2 | 3 | 4 | 5 | 6 | 7 | Favourable |
|  |  | Unappealing | 1 | 2 | 3 | 4 | 5 | 6 | 7 | Appealing |
|  |  | Likeable | 1 | 2 | 3 | 4 | 5 | 6 | 7 | Unlikeable |
|  |  | Pleasant | 1 | 2 | 3 | 4 | 5 | 6 | 7 | Unpleasant |
| Uncle Tobys’ muesli bar | Never heard of it  ☐ | Bad | 1 | 2 | 3 | 4 | 5 | 6 | 7 | Good |
|  |  | Unfavourable | 1 | 2 | 3 | 4 | 5 | 6 | 7 | Favourable |
|  |  | Unappealing | 1 | 2 | 3 | 4 | 5 | 6 | 7 | Appealing |
|  |  | Likeable | 1 | 2 | 3 | 4 | 5 | 6 | 7 | Unlikeable |
|  |  | Pleasant | 1 | 2 | 3 | 4 | 5 | 6 | 7 | Unpleasant |
| Red Bull | Never heard of it  ☐ | Bad | 1 | 2 | 3 | 4 | 5 | 6 | 7 | Good |
|  |  | Unfavourable | 1 | 2 | 3 | 4 | 5 | 6 | 7 | Favourable |
|  |  | Unappealing | 1 | 2 | 3 | 4 | 5 | 6 | 7 | Appealing |
|  |  | Likeable | 1 | 2 | 3 | 4 | 5 | 6 | 7 | Unlikeable |
|  |  | Pleasant | 1 | 2 | 3 | 4 | 5 | 6 | 7 | Unpleasant |
| Carman’s nut bar | Never heard of it  ☐ | Bad | 1 | 2 | 3 | 4 | 5 | 6 | 7 | Good |
|  |  | Unfavourable | 1 | 2 | 3 | 4 | 5 | 6 | 7 | Favourable |
|  |  | Unappealing | 1 | 2 | 3 | 4 | 5 | 6 | 7 | Appealing |
|  |  | Likeable | 1 | 2 | 3 | 4 | 5 | 6 | 7 | Unlikeable |
|  |  | Pleasant | 1 | 2 | 3 | 4 | 5 | 6 | 7 | Unpleasant |
| Go Natural’s nut bar | Never heard of it  ☐ | Bad | 1 | 2 | 3 | 4 | 5 | 6 | 7 | Good |
|  |  | Unfavourable | 1 | 2 | 3 | 4 | 5 | 6 | 7 | Favourable |
|  |  | Unappealing | 1 | 2 | 3 | 4 | 5 | 6 | 7 | Appealing |
|  |  | Likeable | 1 | 2 | 3 | 4 | 5 | 6 | 7 | Unlikeable |
|  |  | Pleasant | 1 | 2 | 3 | 4 | 5 | 6 | 7 | Unpleasant |

1. Regardless of brands, please circle the number which seems most close to how you would describe:

| Nut bars | Bad | 1 | 2 | 3 | 4 | 5 | 6 | 7 | Good |
| --- | --- | --- | --- | --- | --- | --- | --- | --- | --- |
|  | Unfavourable | 1 | 2 | 3 | 4 | 5 | 6 | 7 | Favourable |
|  | Unappealing | 1 | 2 | 3 | 4 | 5 | 6 | 7 | Appealing |
|  | Likeable | 1 | 2 | 3 | 4 | 5 | 6 | 7 | Unlikeable |
|  | Pleasant | 1 | 2 | 3 | 4 | 5 | 6 | 7 | Unpleasant |
| Energy drinks | Bad | 1 | 2 | 3 | 4 | 5 | 6 | 7 | Good |
|  | Unfavourable | 1 | 2 | 3 | 4 | 5 | 6 | 7 | Favourable |
|  | Unappealing | 1 | 2 | 3 | 4 | 5 | 6 | 7 | Appealing |
|  | Likeable | 1 | 2 | 3 | 4 | 5 | 6 | 7 | Unlikeable |
|  | Pleasant | 1 | 2 | 3 | 4 | 5 | 6 | 7 | Unpleasant |

1. Assume that you are looking for a snack/drink and money is not concern, how you would describe your intention to purchase:

|  | Definitely will not purchase | Probably will not purchase | Not sure | Probably will purchase | Definitely will purchase |
| --- | --- | --- | --- | --- | --- |
| V Energy | ☐ | ☐ | ☐ | ☐ | ☐ |
| Uncle Toby’s muesli bar | ☐ | ☐ | ☐ | ☐ | ☐ |
| Red Bull | ☐ | ☐ | ☐ | ☐ | ☐ |
| Carman’s nuts bar | ☐ | ☐ | ☐ | ☐ | ☐ |
| Go Natural’s nut bar | ☐ | ☐ | ☐ | ☐ | ☐ |
| Nut bar *(regardless of brand)* | ☐ | ☐ | ☐ | ☐ | ☐ |
| Energy drink *(regardless of brand)* | ☐ | ☐ | ☐ | ☐ | ☐ |

**Thank you for completing this survey.**
