# Supplementary material for: Exposure to digital marketing enhances young adults’ interest in energy drinks: An exploratory investigation
Source: PLoS One. 2017 Feb 2;12(2):e0171226. doi: 10.1371/journal.pone.0171226 (PMC5289551; doi:10.1371/journal.pone.0171226)
Supplement: S2 Fig — Self-administered survey for experimental group participants after the exposure experiment. (DOCX) [file pone.0171226.s002.docx]

*Participant Number: ____________________ Date: ______________*

**POST-TEST SURVEY**

1. Please circle the number which seems most close to how you would describe:

| Red Bull | Bad | 1 | 2 | 3 | 4 | 5 | 6 | 7 | Good |
| --- | --- | --- | --- | --- | --- | --- | --- | --- | --- |
|  | Unfavourable | 1 | 2 | 3 | 4 | 5 | 6 | 7 | Favourable |
|  | Unappealing | 1 | 2 | 3 | 4 | 5 | 6 | 7 | Appealing |
|  | Likeable | 1 | 2 | 3 | 4 | 5 | 6 | 7 | Unlikeable |
|  | Pleasant | 1 | 2 | 3 | 4 | 5 | 6 | 7 | Unpleasant |
| V Energy | Bad | 1 | 2 | 3 | 4 | 5 | 6 | 7 | Good |
|  | Unfavourable | 1 | 2 | 3 | 4 | 5 | 6 | 7 | Favourable |
|  | Unappealing | 1 | 2 | 3 | 4 | 5 | 6 | 7 | Appealing |
|  | Likeable | 1 | 2 | 3 | 4 | 5 | 6 | 7 | Unlikeable |
|  | Pleasant | 1 | 2 | 3 | 4 | 5 | 6 | 7 | Unpleasant |
| Energy drinks *(regardless of brand)* | Bad | 1 | 2 | 3 | 4 | 5 | 6 | 7 | Good |
|  | Unfavourable | 1 | 2 | 3 | 4 | 5 | 6 | 7 | Favourable |
|  | Unappealing | 1 | 2 | 3 | 4 | 5 | 6 | 7 | Appealing |
|  | Likeable | 1 | 2 | 3 | 4 | 5 | 6 | 7 | Unlikeable |
|  | Pleasant | 1 | 2 | 3 | 4 | 5 | 6 | 7 | Unpleasant |

1. Assume that you are looking for a snack/drink and money is not concern, how you would describe your intention to purchase:

|  | Definitely will not purchase | Probably will not purchase | Not sure | Probably will purchase | Definitely will purchase |
| --- | --- | --- | --- | --- | --- |
| Red Bull | ☐ | ☐ | ☐ | ☐ | ☐ |
| V Energy | ☐ | ☐ | ☐ | ☐ | ☐ |
| Energy drinks *(regardless of brand)* | ☐ | ☐ | ☐ | ☐ | ☐ |

**Thank you for participating in the study.**
