# Supplementary material for: Exposure to digital marketing enhances young adults’ interest in energy drinks: An exploratory investigation
Source: PLoS One. 2017 Feb 2;12(2):e0171226. doi: 10.1371/journal.pone.0171226 (PMC5289551; doi:10.1371/journal.pone.0171226)
Supplement: S5 Fig — Semi-structured interview guide for the control group participants after the exposure experiment. (DOCX) [file pone.0171226.s005.docx]

**SEMI-STRUCUTRED INTERVIEW (Control Group)**

**Introduction**

Thank you for taking part in the study today. Before we start the interview, I’d like to remind you that if there is anything that you’d prefer not to answer, please let me know. In case I miss writing anything down, I’d like to audio record this interview. Is this ok with you? [If not, turn off the recorder].

**SECTION A: Food and beverage preferences**

[Show participants a poster with photos of a range of food and beverage products]

I am going to show you a poster with photos of a variety of food and drinks. Please pick one food or drink item that you would like to have right now?

**SECTION B: Perception of Tested Brands**

1. a) How do you feel about Carman’s and Go Natural after visiting their websites?

b) Would you pay more or less or the same amount of attention to these brands in the future, after looking at their websites?

(Prompt: Pay more or less attention in what ways? eg. Click/avoid their advertisement links? Or specifically look for/avoid their products in shops? Have the websites left you want to learn more or less about these brands?)

c) Have or have the websites not change your views about: i) these brands and ii) their products? In what ways?

(Prompt: Would you more or less likely to try their products?)

**SECTION C:** Experience with digital marketing

1. You’ve just had a look at two websites. Can you please describe the features of the site, including what you thought were good/interesting and what were poor/not so interesting? Let’s start with a) Carman’s website, then b) Go Natural’s website.

**SECTION D: Perception of food and beverage products**

1. What are your views about nut bars? What would you see as the advantages/good points and disadvantages/not so good points about them?

**Prior to the experiment, we also asked you questions about other food and beverage products. Each participant is allocated to answer follow up questions about one of these items. You are allocated to answer questions about energy drinks.**

1. a) When you hear Red Bull, what is the first thing that pops up in your mind?
2. What you hear V Energy, what is the first thing that pops up in your mind?
3. How do you feel about Red Bull and V Energy? (only if participant is aware of these brands)
4. Many young people consume energy drinks. What are your views about them? What would you see as the advantages/good points and disadvantages/not so good points about them?

(Prompt: Have you ever had energy drinks before? What prompt you to drink these drinks? When did you first try energy drinks? In the survey you mentioned that you drink energy drinks, how often? Do friends/ families drink energy drinks? In what occasions? Have you ever mixed alcohol with energy drinks?)
